# In Vitro Evaluation of Intestinal Transport and High-Density Fermentation of *Lactobacillus acidophilus*

**DOI:** 10.3390/metabo13101077

**Published:** 2023-10-13

**Authors:** Xin Su, Bilige Menghe, Heping Zhang, Wenjun Liu

**Affiliations:** 1 Key Laboratory of Dairy Biotechnology and Engineering, Ministry of Education, Inner Mongolia Agricultural University, Hohhot 010018, China; xinsu@emails.imau.edu.cn (X.S.); mhblg@imau.edu.cn (B.M.); 90066@emails.imau.edu.cn (H.Z.); 2 Key Laboratory of Dairy Products Processing, Ministry of Agriculture and Rural Affairs, Inner Mongolia Agricultural University, Hohhot 010018, China; 3 Inner Mongolia Key Laboratory of Dairy Biotechnology and Engineering, Inner Mongolia Agricultural University, Hohhot 010018, China; 4 Collaborative Innovative Center of Ministry of Education for Lactic Acid Bacteria and Fermented Dairy Products, Inner Mongolia Agricultural University, Hohhot 010018, China

**Keywords:** *Lactobacillus acidophilus*, intestinal transport in vitro, high-density fermentation, transcriptomics

## Abstract

*Lactobacillus acidophilus* strains have limiting factors such as low cell density and complex nutritional requirements in industrial production, which greatly restricts their industrial application. In this study, fermentation conditions for *L. acidophilus* were optimized and transcriptomic analysis used to understand growth mechanisms under high-density fermentation conditions. We found that *L. acidophilus* IMAU81186 has strong tolerance to gastrointestinal juice. In addition, its optimal culture conditions were 3% inoculum (*v*/*v*); culture temperature 37 °C; initial pH 6.5; and medium composition of 30.18 g/L glucose, 37.35 g/L soybean peptone, 18.68 g/L fish peptone, 2.46 g/L sodium citrate, 6.125 g/L sodium acetate, 2.46 g/L K_2_HPO_4_, 0.4 g/L MgSO_4_·7H_2_O,0.04 g/L MnSO_4_·5H_2_O, 0.01 g/L serine, and 0.3 g/L uracil. After optimization, viable counts of IMAU81186 increased by 7.03 times. Differentially expressed genes in IMAU81186 were analyzed at different growth stages using transcriptomics. We found that a single carbon source had limitations in improving the biomass of the strain, and *ter*P and *bfr*A were significantly down-regulated in the logarithmic growth period, which may be due to the lack of extracellular sucrose. After optimizing the carbon source, we found that adding 12 g/L sucrose to the culture medium significantly increased cell density.

## 1. Introduction

Probiotics are microorganisms that play a beneficial role in human health. As early as 1899 [1], anaerobic culture was used to isolate beneficial *Bifidobacterium* species from the human body, and probiotics were found for the first time. With continued discovery of probiotics and increased understanding, their utilization has increased. There are many recognized probiotic strains of *Lactobacillus acidophilus*, such as NCFM and La−05 [2,3]. Most are slender rod-shaped bacteria that appear individually, as pairs or in short chains. The edges of colonies on solid media are rough and irregular, usually distributed in line from the center to each edge, and are transparent. The optimum growth temperature for *L. acidophilus* is 35–42 °C [4], while it cannot grow below 20 °C or above 48 °C. Some *L. acidophilus* is strongly resistant to acid and can grow and reproduce in low pH environments. *L. acidophilus* is widely distributed in animal and human microbial systems and is a common member of the microbial flora of feces, the mouth and the vagina [5,6,7]. When *L. acidophilus* reaches a certain level, it can effectively regulate the balance of intestinal flora and maintain a healthy host intestinal tract [8,9,10]. At the same time, *L. acidophilus* also relieves lactose intolerance [11], enhances immunity [12], relieves allergies [13], lowers cholesterol [14] and reduces respiratory tract infections [15]. At present, *L. acidophilus* is a probiotic used widely in China and elsewhere in the world [16], and it has been approved as a functional food and an infant food [17]. It is widely used in commercial probiotic products, including cheese, acidophilic milk, yogurt and dietary supplements [18].

To date, most studies on *L. acidophilus* focus on its probiotic properties, fermentation properties and functional products. However, due to low numbers of viable bacteria, end-product inhibition and insufficient nutrition in the production process, its industrial production has been greatly limited. Finding a culture method that can achieve greater biomass of *L. acidophilus* at low cost has become a focus of research worldwide. High-density cell culture of *Lactobacillus* strains is a critical step in producing large-scale set starters directly, and a key challenge at the industrial scale that mainly involves optimizing the composition of media and culture conditions [19]. As early as 1977, Osborne [20] successfully used a dialysis method to achieve high-density culture of a *Lactobacillus* species for the first time. Since then, scholars from all over the world have developed diverse methods for high-density culture of various lactic acid bacterial strains. In 2012, Krzywonos and Eberhar [21] increased cell density of *Lactiplantibacillus plantarum* and the number of viable bacteria (to 1.6 × 10^10^ CFU/mL) by optimizing media composition. In 2014, Elsayed et al. optimized media composition in high-density culture of *Lactobacillus delbrueckii* subsp. *bulgaricus* and the biomass concentration reached 6.08 g/L [22].

Advances in next generation sequencing (NGS) techniques mean that RNA sequencing (RNA-seq) is becoming a potent and cost-effective way of discovering new genes on a large scale, and it is extensively employed for cloning secondary material associated with biosynthesis, identifying new genetic functions and exploring gene functions [23]. Transcript levels have been reported as a breakthrough point in fungal research [24] and the study of strain growth based on transcriptomics has become a key method.

In the Chinese food production industry, there is little basic research on *L. acidophilus*. The main factors that limit development and utilization of *L. acidophilus* include a lack of probiotic strains, problems of low viable count number, high cost and difficulties in preserving developed products. Therefore, screening of *L. acidophilus* for strains with good probiotic function, achieving high-density culture, accelerating growth and obtaining a *L. acidophilus* bacterial powder with high activity and high viable count are the key challenges in its development and utilization. In recent years, there have been few studies on the analysis of gene expression during high-density fermentation of *L. acidophilus*, so it is particularly important to fully understand and reveal the ongoing metabolic mechanisms.

In this study, *L. acidophilus* with potential probiotic characteristics were evaluated. According to the growth characteristics and nutritional requirements of the strain, we determined an optimal proliferation medium, culture conditions and high-density fermentation processes most suitable for the strain. Finally, differential gene expression profiles of *L. acidophilus* throughout growth were compared using RNA−seq to determine the metabolic mechanism of growth during high-density fermentation.

## 2. Material and Methods

### 2.1. Test Strain

Test strains of *L. acidophilus* were provided by the Key Laboratory of Dairy Biotechnology and Engineering Education, Inner Mongolia Agricultural University. The test strains were isolated from Tibet on horse feces, human feces or koumiss. The control strain was a commercial probiotic (NCFM).

### 2.2. Methods

#### 2.2.1. Preparation of Bacterial Strains

*L. acidophilus* strains that had been stored at −80 °C were inoculated into 5 mL MRS broth (Oxoid, Thermo Fisher Scientific Inc., Basingstoke, UK), cultured at 37 °C, transferred into 40 mL MRS broth at a volume ratio of 2% (*v*/*v*) and cultured for two generations at 37 °C, each for 24 h. Material from each generation was thoroughly washed with PBS (buffered solution) to obtain a pure culture of bacteria and then fully mixed with skimmed milk as a protectant.

#### 2.2.2. Preliminary Selection of *L. acidophilus*

##### Transit Tolerance in Gastrointestinal Juices

Transit tolerance in gastrointestinal juices was tested as described previously [25], activated *L. acidophilus* strains, each with two generations, were washed with sterile PBS (normal saline) to prepare bacterial suspensions. Suspensions were added to artificial gastric juice (Sigma−Aldrich, St. Louis, MI, USA) to achieve a 10% inoculation rate and cultured at 37 °C for 3 h; viable bacteria cell counts were determined in samples taken at 0 h and 3 h. The artificial gastric juice containing bacteria that had been digested for 3 h was added to artificial intestinal juice (Sigma−Aldrich, St. Louis, MI, USA) to achieve a 10% inoculation rate, and the total viable counts determined after 4−8 h incubation.

##### Bile Salt Tolerance of *L. acidophilus*

Bile tolerance was tested as described previously [26]. Evaluated strains were inoculated into either MRS broth containing 3% bovine bile salt (Sigma-Aldrich, St. Louis, MI, USA) to achieve an inoculation rate of 2%, or MRS broth without bovine bile salt at the same rate as the control. Inoculations were cultured at a constant temperature of 37 °C and the cell density measured every 1 h until cell density of the test group and the control group had increased by more than 0.3 units. We calculated the difference in the time to achieve this increase between the treatment and control (the lag time).

#### 2.2.3. High Density Culture

##### Optimization of Static Culture Conditions

The effects of temperature, pH and inoculum size on growth of *L. acidophilus* were studied using an orthogonally designed test in MRS broth, and the best static culture conditions determined by comparing bacterial growth.

##### Optimization of Culture Medium Composition

MRS broth was used as the basic medium but various proportions or different carbon sources (raffinose, stachyose, D-glucosamine, β-methyl-galactoside, dihydroxy acetone, acetyl glucosamine, glucose, lactose, mannose, xylose, ribose, lyxose) and nitrogen sources (soybean peptone, beef extract, beef peptone, beef dip powder, tryptone, fish peptone, proteose peptone, casein peptone, yeast extract, yeast peptone, yeast, liver extract powder (beef), compound nitrogen source (MRS)) were used to replace the original carbon and nitrogen sources [27]. Different types and proportions of buffer salt systems ((C_6_H_8_O_7_/C_6_H_5_Na_3_O_7_), (Na_2_HPO_4_/NaH_2_PO_4_), (Na_2_HPO_4_/C_6_H_8_O_7_), (KH_2_PO_4_/NaOH), (C_6_H_5_Na_3_O_7_/CH_3_COONa/K_2_HPO_4_)), trace elements (MgSO_4_·7H_2_O, MnSO_4_·5H_2_O, FeSO_4_·7H_2_O, CuSO_4_·5H_2_O, ZnSO_4_·7H_2_O) and growth factors (valine, arginine, alanine, methionine, proline, glutamic acid, serine, histidine, asparagine, lysine, leucine and threonine, adenine, urine purine, cytosine, thymine, V_B1_, V_B2_, V_B5_, V_B6_, V_C_, and L−cysteine hydrochloride) were evaluated in the same way. The optimum medium for *L. acidophilus* growth was determined by measuring cell density in these different media combinations.

##### Optimization of High-Density Fermentation Process

As in the above experiments, high−density fermentation conditions (constant pH: 5/5.5/6/6.5; neutralizing agent: NaOH, NH_3_·H_2_O, Na_2_CO_3_) of *L. acidophilus* were optimized by evaluating single factors in 5 L liquid fermenter. By counting viable bacteria in fermentation broth, the optimal high-density fermentation process was determined.

##### Growth Curve of *L. acidophilus*

Samples were taken every 2 h during high-density fermentation, and cell density measured. The growth curve was drawn according to cell density, the number of viable bacteria and pH value. Sampling points were the beginning of fermentation (T0), the beginning of the logarithmic phase (T4), the end of the logarithmic phase (T10) and the stable phase (T14). Suspensions were centrifuged at 4000 r/min for 10 min and the bacterial cells collected. Cells were cleaned with sterile PBS (normal saline) twice, and quickly cooled in liquid nitrogen prior to storage in a −80 °C refrigerator for later use in transcriptomics research.

#### 2.2.4. Transcriptional Studies at Different Growth Stages

##### RNA Extraction

TRIzol reagent (Invitrogen, CA, USA) was used to extract total RNA from bacteria following the manufacturer’s instructions. A transcriptome sequencing (RNA−seq) library was prepared with the Illumina TruSeq RNA sample preparation kit (Illumina, San Diego, CA, USA). The paired-end RNA−seq sequencing library was sequenced with the Illumina HiSeq X 10 (2150 bp read length). Raw sequences were processed using the Illumina GA pipeline (version 1.6) to obtain paired-end reads of 150 bp.

##### Transcriptome Analyses

First, low-quality bases were filtered to obtain clean data. The clean reads were then compared with the reference genome sequence of *L. acidophilus* IMAU81186 using Bowtie 2 [28]. Differentially expressed genes were identified by RSEM (http://deweylab.biostat.wisc.edu/rsem/, accessed on 12 May 2020), Kallisto (https://pachterlab.github.io/kallisto/, accessed on 17 May 2020) and Salmon (https://combine-lab.github.io/salmon/, accessed on 3 June 2020). Differential gene expression was calculated by the EdgeR and DESeq2 packages in R (version 4.2.1) [29]. Genes with *p* values of <0.05 and FCs of >2 were considered significantly upregulated, while those with *p* values of <0.05 and FCs of <0.5 were considered significantly downregulated.

#### 2.2.5. Statistical Analysis

All statistical and visual analyses were mainly performed using R (version R (4.2.1)) software. Wilcoxon and Kruskal−Wallis tests were carried out with ggpubr package in R software, and the differences between different groups were calculated using IBM SPSS Statistics 20. The design of the response surface was analyzed by Design Expert (V13.0.5.0). Origin (2018) software was used to fit the principal component equation for the culture medium and draw the chart.

## 3. Results and Discussion

### 3.1. Gastrointestinal Fluid Tolerance and Bile Salt Tolerance

**Figure 1:**
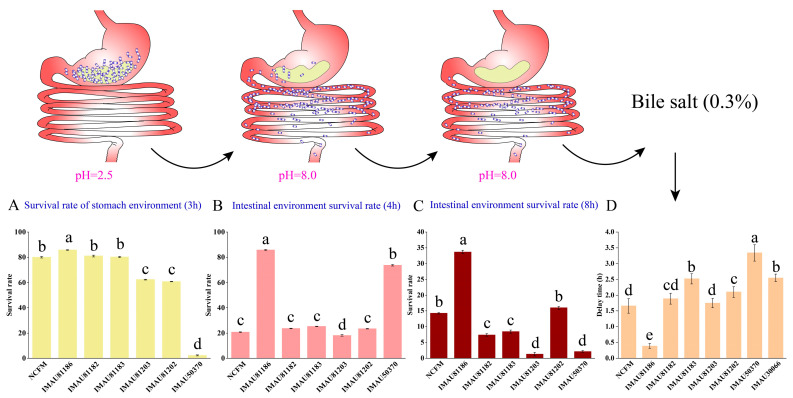
Gastrointestinal environment and bile salt tolerance. (**A**) Survival rate of the strain after 3 h digestion in simulated gastric fluid. (**B**) Survival rate of the strain after 4 h digestion in simulated intestinal fluid. (**C**) The survival rate of bacterial strains after 8 h digestion in simulated intestinal fluid. (**D**) Bile salt tolerance of the strain was determined (the shorter the delay time, the stronger the tolerance of the strain). Error bars represent standard error of the mean. The different letters above the bars in the figure indicate that there are significant differences between the data (*p* < 0.05).

Digestion time of food in the stomach environment at pH 2.5 is about 3 h, and then it is further digested in the small intestine at pH 8.0. In this experiment, eight strains of *L. acidophilus* were selected from the resource bank of the Inner Mongolia Agricultural University; *L. acidophilus* NCFM was used as the control strain (Figure 1). Strain IMAU81186 has the strongest tolerance to gastric juice, with a survival rate is 85.76% after being digested for 3 h. It was then inoculated into intestinal juice, and the survival rate after 8 h of digestion was 33.67%, which was significantly higher than that of the control strain, NCFM (*p* < 0.05). Moreover, the comprehensive gastrointestinal juice survival rate showed that IMAU81186 has the highest overall tolerance. A suitable concentration of bile salt can increase cell wall permeability to a certain extent, so that alcohol can better enter the cell and reduce the body’s cholesterol concentration [30]. This experiment determined the tolerance of *L. acidophilus* to bile salts at a concentration of 0.3%. The delay time of the test strain, IMAU81186, was 0.39 h, which was the lowest (Figure 1). Some studies have shown that *L. acidophilus* NCEM has strong bile tolerance and can survive in the intestine for a long time, ensuring it has a probiotic function; it also has significant cholesterol-lowering ability [31]. Based on the results described above, *L. acidophilus* IMAU81186 was selected for further study. Cook et al. showed that acid tolerance of LAB is related to induction of H^+^-ATPase activity [32]. In addition, Conway et al. found that survival rates of LAB in PBS solution were relatively low compared to survival rates in gastric juice, which may be because components of gastric juice have a protective effect on bacterial cells [33].

### 3.2. Optimization of Culture Conditions

An orthogonal design was used to determine the best static culture conditions for *L. acidophilus* IMAU81186 (Table S2). Through range analysis of three factors, we concluded that the order of influence of static culture conditions on cell density of *L. acidophilus* IMAU81186 was pH > temperature > inoculation volume. The best static culture conditions were pH 6.5, temperature 35 °C, and 3% inoculum (*v*/*v*).

### 3.3. Optimization of Media

#### 3.3.1. Carbon and Nitrogen Source Optimization

**Figure 2:**
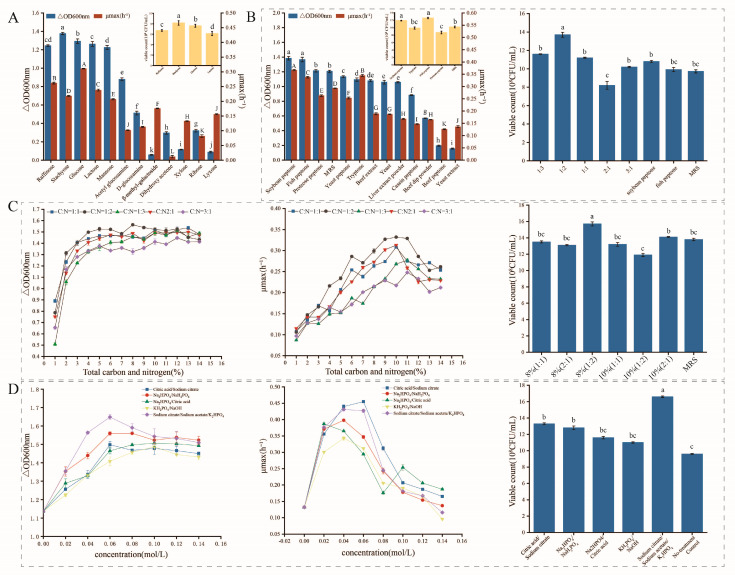
(**A**) Effects of different carbon sources on growth of *L. acidophilus* IMAU81186 (blue is △OD_600nm_, red is μmax (h^−1^), yellow is viable bacteria number). (**B**) Effects of different nitrogen sources on growth of *L. acidophilus* IMAU81186 (left), viable bacteria count (right). (**C**) Effects of C/N ratio and C/N total on growth of *L. acidophilus* IMAU81186: △OD_600nm_ (left), μmax (h^−1^) (middle), viable bacteria number (right). (**D**) Effects of different buffer salts on growth of *L. acidophilus* IMAU81186: △OD_600nm_ (left), μmax (h^−1^) (middle), viable bacteria number (right). Error bars represent standard error of the mean. The different letters above the bars in the figure indicate that there are significant differences between the data (*p* < 0.05).

An appropriate medium is essential for cell growth and synthesis of target metabolites. Twelve carbon sources were evaluated for their suitability for growth of *L. acidophilus* IMAU81186, according to Berger’s bacterial handbook. Based on cell density and maximum specific growth rate analysis, *L. acidophilus* IMAU81186 had the greatest capacity for utilization of stachyose (Figure 2A), followed by glucose, raffinose and lactose. The number of viable bacteria produced was greatest when the carbon source was stachyose (8.16 ± 0.42 × 10^8^ CFU/mL), followed by glucose (7.82 ± 0.28 × 10^8^ CFU/mL). According to the principle of low cost and high yield of high-density fermentation, stachyose is not suitable for industrial batch production due to its high price. Therefore, glucose was selected as the best carbon source for *L. acidophilus* IMAU81186. Our results are similar to those of Pedram et al. [34], who also suggest that adding a certain amount of glucose to the culture medium can significantly improve the biomass of a strain.

Nitrogen is an essential factor in the biosynthesis of nucleic acids or proteins [35]. The type and concentration of nitrogen source have been reported to influence carbon transport to cell biomass [36]. Out of 12 nitrogen sources, *L. acidophilus* IMAU81186 utilization of soybean peptone and fish peptone was significantly higher than its utilization of the other nitrogen sources and control (*p* < 0.05) (Figure 2B); this was followed by peptone group, tryptone group and MRS nitrogen sources. Combinations of nitrogen sources are more conducive to growth of LAB [37]; hence, we compared growth in compound media. When the ratio of soybean peptone to fish peptone was 1:2, the viable count of *L. acidophilus* IMAU81186 reached 1.37 ± 0.26 × 10^9^ CFU/mL, which was 1.4 times greater than before optimization. Therefore, the best nitrogen source for this strain was the compound nitrogen source of soybean peptone and fish peptone (ratio = 1:2).

Based on the above experimental results, the effects of different C/N ratios and total C/N on the growth of the strain were analyzed. As shown in Figure 2C, when the total C/N ratio is 8% and the C/N ratio is 1:2, *L. acidophilus* IMAU81186 can reach the highest biomass.

#### 3.3.2. Buffer Salt Optimization

Based on the optimal pH value of *L. acidophilus* IMAU81186, five buffer salt systems were selected for comparison. When 0.06 mol/L C_6_H_5_Na_3_O_7_/CH_3_COONa/K_2_HPO_4_ was added as the buffer salt system, the cell density, maximum specific growth rate, and number of viable bacteria of *L. acidophilus* IMAU81186 reached a maximum (Figure 2D). The number of viable bacteria was 1.66 ± 0.09 × 10^9^ CFU/mL, which was significantly higher than the other experimental groups (*p* < 0.05).

#### 3.3.3. Growth Factor Optimization

**Figure 3:**
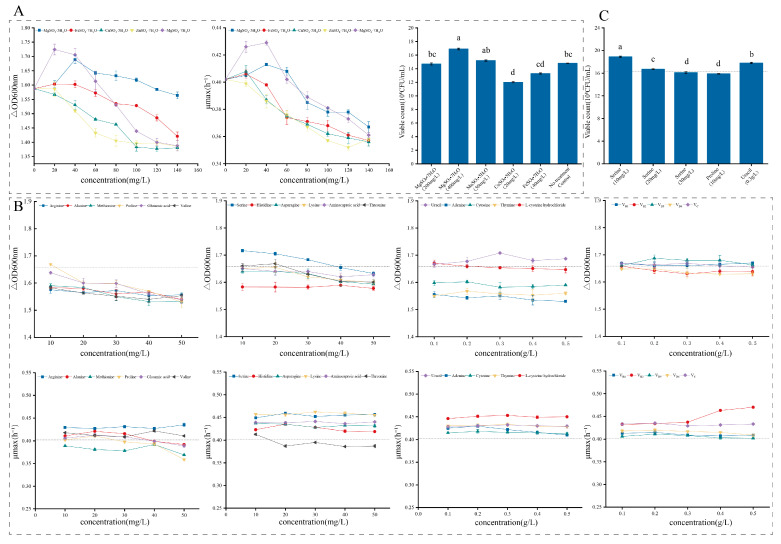
(**A**) Influence of trace elements on growth of *L. acidophilus* IMAU81186 (different colors represent different trace elements). (**B**) Influence of amino acids, nucleotides and vitamins on growth of *L. acidophilus* IMAU81186. (**C**) Influence of growth factors on number of live bacteria of the strain. Error bars represent standard error of the mean. The different letters above the bars in the figure indicate that there are significant differences between the data (*p* < 0.05).

An appropriate concentration of trace elements not only can increase the density of LAB, it can also improve acid and alkali resistance, temperature resistance and antibacterial ability [38,39]. In this experiment, five commonly used trace elements were evaluated at different concentration gradients. It was found that appropriate concentrations of MgSO_4_·7H_2_O, MnSO_4_·5H_2_O and FeSO_4_·7H_2_O increase cell density, but as the concentration increased, they all inhibited cell growth (Figure 3A). Combined with the analysis of multiple indicators, it was found that MgSO_4_·7H_2_O at a concentration of 400 mg/L and MnSO_4_·7H_2_O at a concentration of 40 mg/L significantly improved growth characteristics compared with other growth factors (*p* < 0.05); viable cell counts were 1.69 ± 0.13 × 10^9^ CFU/mL and 1.52 ± 0.11 × 10^9^ CFU/mL, respectively. This may be because Mg^2+^ promotes ATP formation during growth of LAB cells [38]; many studies have also shown that Mg^2+^ is an essential trace element for growth and reproduction of LAB, resulting in significant biomass production [40]. As early as 1985, Amouzou [39] studied the effect of Mg^2+^ on biomass of *Streptococcus thermophilus* and *Streptococcus lactis*, and found that both biomass and growth rate increased.

LAB have strict requirements for nutrients. Growth and reproduction require a certain quantity of external growth factors. Most LAB require vitamins, purine and pyrimidines [41]. For this reason, the effects of different types and concentrations of growth factors on *L. acidophilus* IMAU81186 were studied. This strain varied in its ability to use different growth factors (Figure 3B). Compared with the control group without growth factors, 10–30 mg/L serine and 0.3 g/L uracil resulted in significant increases in growth (*p* < 0.05); 10 mg/L proline also has a slight positive effect on growth. Most of the other amino acids had no effect or even restricted growth. Analyzing the number of viable cells showed that 10 mg/L serine and 0.3 g/L uracil resulted in a significant increase in the number of viable bacteria (*p* < 0.05) to 1.89 ± 0.13 × 10^9^ CFU/mL and 1.77 ± 0.12 × 10^9^ CFU/mL, respectively (Figure 3C). Overall, serine at 10 mg/L and uracil at 0.3 g/L were selected as enrichment factors in the medium of *L. acidophilus* IMAU81186.

#### 3.3.4. Response Surface Optimization

The response surface method has been used widely to optimize culture media [42,43]. We used Design Expert software to optimize main culture medium components (Tables S3 and S4). The equation of quadratic polynomial regression model is △OD = 10.72 + 1.48A + 0.90B + 0.44C − 0.68AB − 0.21AC − 0.008BC − 2.14A^2^ − 2.28B^2^ − 2.76C^2^, indicating a good fit with the experiment and the capability to predict growth of *L. acidophilus* IMAU81186.

**Figure 4:**
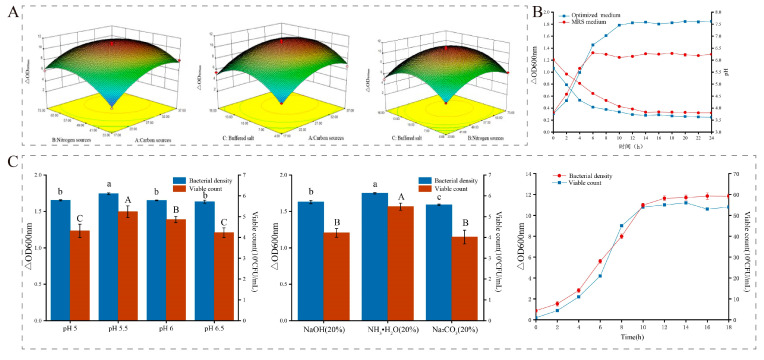
(**A**) Response surface optimization results: carbon and nitrogen source response surface diagram (left), carbon source and buffer salt response surface diagram (middle), nitrogen source and buffer salt response surface diagram (right). (**B**) Comparison of growth curves before and after optimization (blue is optimal medium, red is MRS medium). (**C**) High-density fermentation process optimization results: fermenter constant pH optimization results (left), fermenter neutralizer optimization results (middle), high-density fermentation growth curve (right). Error bars represent standard error of the mean. The different letters above the bars in the figure indicate that there are significant differences between the data (*p* < 0.05).

The two-dimensional contour and three-dimensional map obtained showed that the number of carbon and nitrogen sources significantly affects the cell density of *L. acidophilus* IMAU81186 (Figure 4). However, the curvature between the carbon source and the buffer salt, and between the nitrogen source and the buffer salt was very small, suggesting that the interactions were not significant.

The optimum quantities of carbon source, nitrogen source and buffer salt obtained by response surface methodology were 30.18 g/L, 56.03 g/L and 11.04 g/L, respectively. Cell density predicted by the model was 11.0411. To verify the reliability of the prediction, *L. acidophilus* IMAU81186 was cultured at 37 °C for 24 h with optimized medium composition, and the final cell density was 10.953, which was not significantly different from the predicted value (*p* > 0.05). Therefore, we consider that response surface methodology was accurate in predicting the optimal composition of LAB culture medium.

Based on experimental results, the optimized medium for static culture was glucose 30.18 g/L, soybean peptone 37.35 g/L, fish peptone 18.68 g/L, sodium citrate 2.46 g/L, sodium acetate 6.125 g/L, K_2_HPO_4_ 2.46 g/L, MgSO_4_·7H_2_O 0.4 g/L, MnSO_4_·5H_2_O 0.04 g/L, serine 0.01 g/L and uracil 0.3 g/L.

To analyze growth changes in *L. acidophilus* IMAU81186, optimized medium components were used for static culture of the strain, and MRS medium was used as the control group. The cell density of the optimized medium was significantly higher than that of MRS medium. The logarithmic growth period was significantly prolonged; the pH decreased faster than that of MRS medium (Figure 4B). After optimization, the number of viable bacteria reached 2.14 ± 0.24 × 10^9^ CFU/mL, demonstrating that the optimized medium was more suitable for growth and reproduction of *L. acidophilus* IMAU81186 than MRS.

High-density fermentation of *L. acidophilus* is important for industrial upscaling. There have been several studies on high-density culture of *L. acidophilus* strains. Meena [41] et al. found that fructose was the carbon source that *L. acidophilus* NCDC 14 had the greatest capacity to utilize. Park [44] studied the growth factors that promote growth and reproduction of *L. acidophilus* 333 and found that growth required participation of V_B1_, V_B3_ and V_B5_. Several studies have shown that growth and reproduction of *L. acidophilus* requires participation of various essential amino acids, and *L. acidophilus* strains vary significantly in their capacities to utilize nutrients, so it is necessary to optimize medium components for each strain.

### 3.4. High-Density Fermentation Technology

LAB produces lactic acid during growth and reproduction so its growth environment is acidic, which can cause damage and slow growth. Therefore, regulating the pH of the culture environment and adding appropriate neutralizers can improve growth and metabolism and increase biomass. In the high-density fermentation optimization process, we found that when pH = 5.5 cell density and viable counts of *L. acidophilus* IMAU81186 were significantly higher than in other experimental groups (*p* < 0.05) (Figure 4C). When 20% NH_3_·H_2_O. was added, biomass yield was also significantly improved. Therefore, optimal fermentation process conditions were set as pH = 5.5 and the neutralizer was 20% NH_3_·H_2_O.

The fermentation experiment in 5 L automatic mechanical stirring fermenters used the optimized culture medium from previous experiments; cell density and the number of viable bacteria in the fermentation broth were monitored in real time every 2 h. The viable count of *L. acidophilus* IMAU81186 in the high-density fermenter reached 5.5 ± 0.43 × 10^9^ CFU/mL, which is 7.03 times higher than achieved using the previous optimization (Figure 4C).

### 3.5. Transcriptional Analysis

#### 3.5.1. Identification of Differentially Expressed Genes at Various Growth Stages

**Figure 5:**
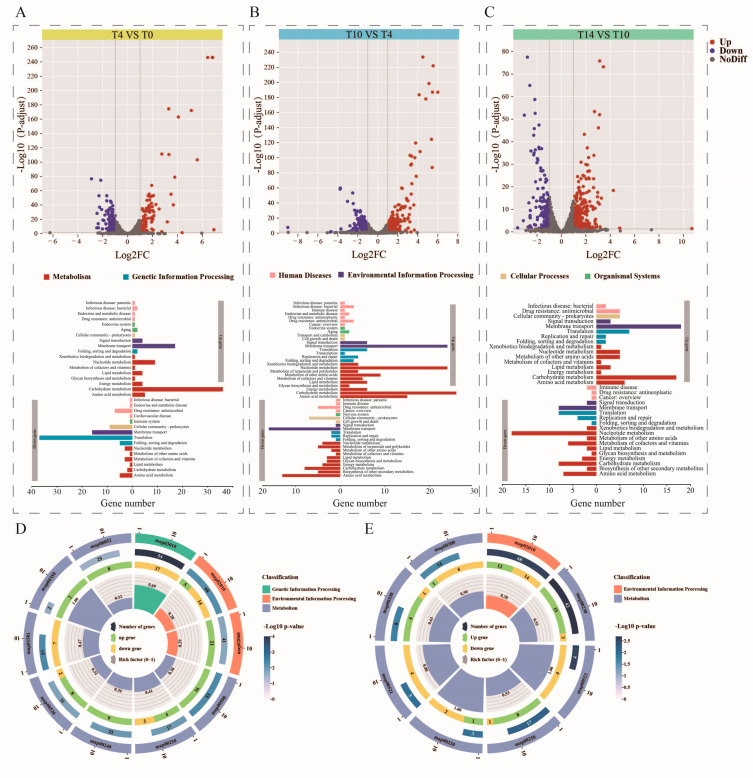
(**A**) T4 vs. T0 DGE summary results volcano map (top) and KEGG annotation analysis (bottom); (**B**) T10 vs. T4; (**C**) T14 vs. T10. Enrichment analysis results for DGEs at (**D**) T4 vs. T0; (**E**) T10 vs. T4. Different biological pathways are shown in different colors.

Differentially expressed genes (DEGs) across different growth stages were identified based on TPM by applying a cutoff of *p* < 0.05. A total of 246 genes were differentially expressed between T0 and T4. Among these, 118 up-regulated genes (48% of all significant DEGs) were involved in membrane transport, carbohydrate metabolism and nucleotide metabolism. Furthermore, 128 down-regulated genes (52% of all significant DEGs) were involved in translation, membrane transportation and antimicrobial drug resistance (Figure 5A). There were 446 significant DEGs between T4 and T10 samples, of which 224 up-regulated genes (50.2%) were related to nucleotide metabolism, membrane transport, carbohydrate metabolism and amino acid metabolism; and 222 down-regulated genes (49.8%) were related to membrane transport and amino acid metabolism (Figure 5B). There were 390 DEGs identified between T10 and T14, with 230 up-regulated (carbohydrate metabolism) and 160 down-regulated (nucleotide metabolism) genes (Figure 5C). There were 26 DEGs shared among the three groups.

The numbers of specific DEGs in T10 vs. T4 and T14 vs. T10 were remarkably greater than those in T4 vs. T0, indicating involvement of complex developmental events in the logarithmic phase of *L. acidophilus* growth. A large proportion of DEGs (99 genes) were common between T10 vs. T4 and T14 vs. T10, suggesting that they were specifically involved in the developmental processes in the logarithmic phase.

To further study the growth mechanism of strains in different periods, we enriched and analyzed DEGs in Kyoto Encyclopedia of Genes and Genomes (KEGG). Between the adaptive phase (T0) and the initial logarithmic phase (T4), ribosome, ABC transporters and starch and sucrose metabolism were represented by the greatest number of DEGs with the minimum *p* value (*p* < 0.05) (Figure 5D). Between the initial logarithmic phase (T4) and the end of the logarithmic phase (T10), a large number of DEGs were significantly enriched in purine metabolism, ABC transporters, and alanine, aspartate and glutamate metabolism (Figure 5E). Genes with the minimum Q value between the end of the logarithmic phase (T10) and the stationary phase (T14) were involved in purine metabolism.

#### 3.5.2. Characterization of Functional Genes in *L. acidophilus* at Different Growth Phase by KEGG Pathway Analysis

**Figure 6:**
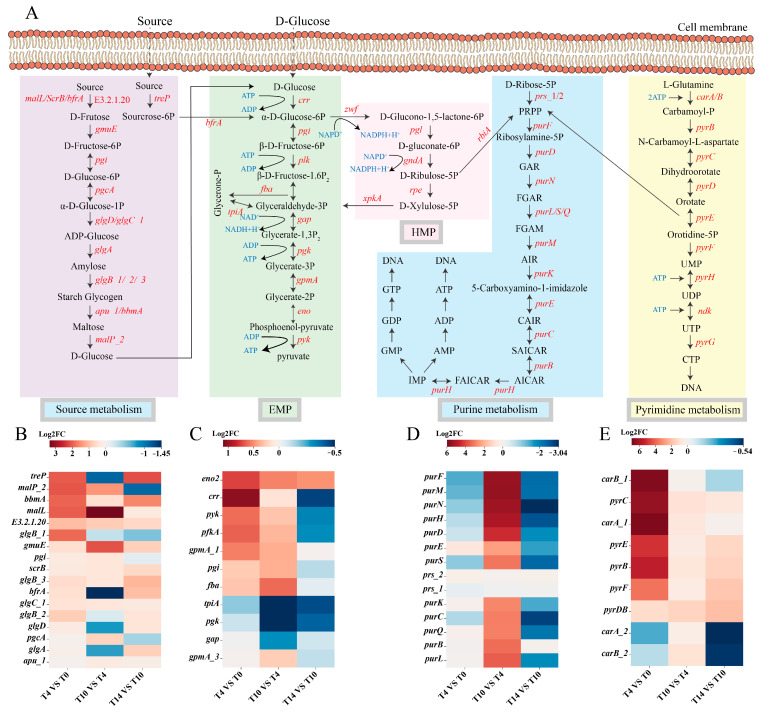
(**A**) Diagram of sucrose metabolism, glycolysis, the pentose phosphate pathway, purine metabolism and the pyrimidine metabolism pathway. (**B**) Heat map, (**C**) glycolysis pathway, (**D**) purine metabolism, (**E**) pyrimidine metabolism of sucrose metabolism-related genes at different growth stages.

Carbohydrate metabolism and nucleotide metabolism pathways were analyzed (Figure 6A). Purine nucleotide was the key metabolite in the cell; it is both a component of DNA and RNA, and has the energy carriers, ATP and GTP, involved in various metabolic processes in the cell. The expression of its key genes is as shown in Figure 6D. When the adaptive phase (T0) entered the initial logarithmic phase (T4), the cell was adapting to the nutritional environment, in the growth stage, and not involved in much DNA synthesis, so its expression level had not significantly increased. However, when the strain entered the end of the logarithmic phase (T10), all genes except *prs*_1/2 were up-regulated by more than two-fold. After entering the stationary phase (T14), activity of de novo synthesis of purine was inhibited, and expression of key genes began to decrease significantly, but the PRPP gene *prs*_1/2 was up-regulated to some extent, and *pur*F was down-regulated. We speculate that PRPP synthesized during this period is involved in pyrimidine metabolism and histidine metabolism. The expression level of key genes involved in pyrimidine nucleotide metabolism are shown in Figure 6E, and the gene *car*A_2/B_2 encoding carbamoyl phosphate synthase did not increase significantly when the adaptive phase (T0) entered the initial logarithmic phase (T4), which may be due to the key role of the gene *car*A_1/B_1. At the end of the logarithmic phase (T10), all genes were up-regulated to a small extent, while at the end of the stationary phase (T14), *car*A/B genes were down-regulated to some extent due to the lack of glutamic acid.

Expression levels of key genes in the sugar metabolism pathway were analyzed by thermogram. Expression levels of glycolysis-related genes remained high during the whole growth period, and the expression level of *crr* continued to increase in the adaptive phase (T0) and logarithmic phase (T10−T4), and then decreased after entering the stationary phase (T14) (Figure 6C). There are two reasons for this phenomenon. The first is that the PTS enzyme was inhibited, and the second is that glucose in the culture medium has been completely consumed by the strains in logarithmic phase (T10−T4). When the body cannot use glucose, the PTS operon involved in regulation activates adenylate cyclase and consumes ATP, resulting in a decrease in ATP and slow growth. Moreover, we found that expression of all genes in the glycolytic pathway slowed down when they entered the end of the logarithmic phase (T10), and all were down-regulated to different degrees when they entered the stationary phase (T14). This may be related to the carbon source content in the culture environment, which also reflects the limitations of carbon source optimization in our optimization process. At the same time, analysis showed that sucrose metabolism could synthesize intermediate metabolites of glycolytic pathway, which can promote aerobic respiration (Figure 6B). The overall expression level of sucrose metabolism was low because an appropriate amount of sucrose was not provided outside the cell. The gene involved in extracellular sucrose decomposition is *ter*P, which was significantly down-regulated from the initial logarithmic phase (T4) to the end of the logarithmic phase (T10); all genes involved in sucrose metabolism were at a high expression level during the initial logarithmic phase (T4). After the end of the logarithmic phase (T10), most genes were not significantly down-regulated, while *ter*P and *bfr*A, the genes involved in metabolism of extracellular sucrose, were down-regulated to varying degrees. Therefore, we speculate that the biomass of *L. acidophilus* IMAU81186 can be effectively increased using the compound carbon source of sucrose and glucose.

**Figure 7:**
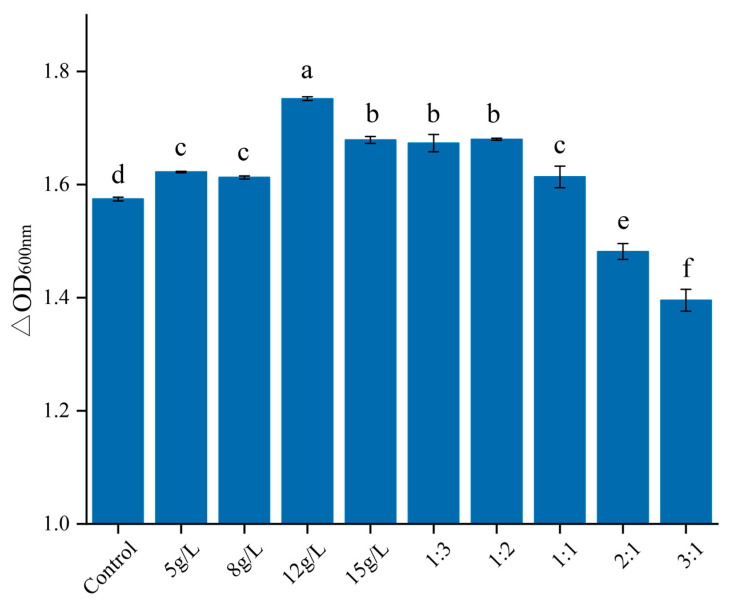
Determination of cell density achieved when different carbon sources were used in the medium. The ratio is sucrose: glucose. Control is the optimal medium without sucrose. Error bars represent standard error of the mean. The different letters above the bars in the figure indicate that there are significant differences between the data (*p* < 0.05).

Based on the experimental results, carbon sources in the culture medium were further optimized. By combining transcriptomics with pure culture, the optimized culture medium components obtained in this experiment were glucose 30.18 g/L, sucrose 12 g/L, soybean peptone 37.35 g/L, fish peptone 18.68 g/L, sodium citrate 2.46 g/L, sodium acetate 6.125 g/L, K_2_HPO_4_ 2.46 g/L, MgSO_4_·7H_2_O 0.4 g/L, MnSO_4_·5H_2_O 0.04 g/L, serine 0.01 g/L and uracil 0.3 g/L. When 12g/L sucrose was added to the culture medium, cell density significantly increased (Figure 7).

## 4. Conclusions

In this study, the gastrointestinal fluid tolerance of *L. acidophilus* was evaluated in vitro, and the strain IMAU81186 with strong tolerance was obtained. The culture conditions, medium components and high-density fermentation technology were optimized. The results showed that the number of viable bacteria of *L. acidophilus* IMAU81186 increased by 7.03 times after optimization. By analyzing the differentially expressed genes, it was found that *L. acidophilus* IMU81186 could effectively utilize the sucrose provided outside the cell, and the biomass increased significantly when 12 g/L sucrose was added.

## Data Availability

The original data of transcriptome sequencing in this study have been deposited at the National Center of Biotechnology Information (NCBI).

## Supplementary Materials

The following supporting information can be downloaded at: https://www.mdpi.com/article/10.3390/metabo13101077/s1, Table S1. Information about Lactobacillus acidophilus strains evaluated; Table S2. Orthogonal test L16(34) of static culture conditions; Table S3. CCRD design with experimental and predicted cell population of *L. acidophilus* IMAU81186; Table S4. Coefficients and ANOVA for the developed quadratic polynomial model.
