# Supplementary material for: In Vitro Evaluation of Intestinal Transport and High-Density Fermentation of Lactobacillus acidophilus
Source: Metabolites. 2023 Oct 13;13(10):1077. doi: 10.3390/metabo13101077 (PMC10609339; doi:10.3390/metabo13101077)
Supplement: Supplementary file 1 [file metabolites-13-01077-s001.zip › metabolites-2582641-supplementary.pdf]

TABLES

Table S1. Information about *Lactobacillus acidophilus* strains evaluated

Strain reference	Date of isolation	Region from which strain was isolated	Isolation source
IMAU81186	2014	Hongyuan County, Sichuan Province	Tibetan horse faeces
IMAU81182	2014	Hongyuan County, Sichuan Province	Tibetan horse faeces
IMAU81183	2014	Hongyuan County, Sichuan Province	Tibetan horse faeces
IMAU81203	2014	Hongyuan County, Sichuan Province	Tibetan horse faeces
IMAU81202	2014	Hongyuan County, Sichuan Province	Tibetan horse faeces
IMAU50370	2017	Xiangyang of Hubei Province	Adult faeces
IMAU30066	2004	Huocheng County, Xinjiang	Koumiss
NCFM	CK	Commercial probiotic	

Table S2. Orthogonal test L16（34）of static culture conditions

Number	A	B	C	Bacterial density (△ OD600nm)
	Temperature (℃)	pH	Inoculation quantity	
1	1(31 ℃)	1(5.0)	1 (1%)	0.5872
2	1	2 (5.5)	2 (2%)	0.7587
3	1	3 (6.0)	3 (3%)	0.9403
4	1	4 (6.5)	4 (4%)	0.9046
5	2 (35 ℃)	1	2	0.8890
6	2	2	1	0.7975
7	2	3	4	1.0343
8	2	4	3	1.2747
9	3 (39 ℃)	1	3	0.8823
10	3	2	4	0.9023
11	3	3	1	0.9147
12	3	4	2	1.0206
13	4 (43 ℃)	1	4	0.7006
14	4	2	3	0.7892

15	4	3	2	0.8373
16	4	4	1	0.8241
K1	0.8227	0.7648	0.7809	
K2	0.9989	0.8369	0.8764	
K3	0.9549	0.9316	0.9716	
K4	0.7878	1.0060	0.8855	
R	0.2111	0.2412	0.1907	
Factor arrangement		B>A>C		
Horizontal arrangement	K2 > K3 > K1 > K4	K4 > K3 > K2 > K1	K3 > K4 > K2 > K1	
Excellent combination	A2	B4	C3	
Excellent level		$A_2B_4C_3$		

Table S3. CCRD design with experimental and predicted cell population of *L. acidophilus* IMAU81186

Number	A<br>carbon source<br>(g/L)	B<br>nitrogen source<br>(g/L)	C<br>Buffer salt<br>(g/L)	Bacterial density<br>△OD_{600nm}
1	1	1	0	8.2342
2	0	0	0	10.8679
3	-1	0	1	5.4622
4	1	0	1	7.3651
5	0	0	0	9.9427
6	0	-1	1	5.0023
7	1	0	-1	6.6244
8	0	0	0	10.9434
9	-1	0	-1	3.8621
10	-1	-1	0	3.0154
11	0	-1	-1	4.3998

12	-1	1	0	6.0093
13	1	-1	0	7.9416
14	0	0	0	11.2088
15	0	0	0	10.6548
16	0	1	-1	6.383
17	0	1	1	6.9552

Table S4. Coefficients and ANOVA for the developed quadratic polynomial model

Source	Sum of Squares	df	Mean square	F value	P-value
Model	109.19	9	12.13	44.36	< 0.0001
A	17.45	1	17.45	63.82	< 0.0001
B	6.52	1	6.52	23.84	0.0018
C	1.54	1	1.54	5.65	0.0491
AB	1.82	1	1.82	6.67	0.0363
AC	0.18	1	0.18	0.68	0.4383
BC	0.00	1	0.00	0.00	0.9777
A^2	19.28	1	19.28	70.51	< 0.0001
B^2	21.95	1	21.95	80.28	< 0.0001

C^2	31.96	1	31.96	116.87	< 0.0001
Residual	1.91	7	0.27		
Lack of Fit	1.00	3	0.33	1.44	0.3552
Pure Error	0.92	4	0.23		
Cor Total	111.10	16			
	R^2=0.9828			R_{adj}^2=0.9606	
